# Asymmetric Reproductive Barriers and Gene Flow Promote the Rise of a Stable Hybrid Zone in the Mediterranean High Mountain

**DOI:** 10.3389/fpls.2021.687094

**Published:** 2021-08-25

**Authors:** Mohamed Abdelaziz, A. Jesús Muñoz-Pajares, Modesto Berbel, Ana García-Muñoz, José M. Gómez, Francisco Perfectti

**Affiliations:** ^1^Departamento de Genética, Facultad de Ciencias, Campus Fuentenueva, Universidad de Granada, Granada, Spain; ^2^Laboratório Associado, Plant Biology, Research Centre in Biodiversity and Genetic Resources, Centro de Investigação em Biodiversidade e Recursos Genéticos, Universidade Do Porto, Campus Agrário de Vairão, Fornelo e Vairão, Portugal; ^3^Research Unit Modeling Nature, Universidad de Granada, Granada, Spain; ^4^Departamento de Ecología Funcional y Evolutiva, Estación Experimental de Zonas Áridas, Consejo Superior de Investigaciones Científicas, Almeria, Spain

**Keywords:** hybridization, *Erysimum mediohispanicum*, *Erysimum nevadense*, Sierra Nevada, phenotype, reproductive isolation

## Abstract

Hybrid zones have the potential to shed light on evolutionary processes driving adaptation and speciation. Secondary contact hybrid zones are particularly powerful natural systems for studying the interaction between divergent genomes to understand the mode and rate at which reproductive isolation accumulates during speciation. We have studied a total of 720 plants belonging to five populations from two *Erysimum* (Brassicaceae) species presenting a contact zone in the Sierra Nevada mountains (SE Spain). The plants were phenotyped in 2007 and 2017, and most of them were genotyped the first year using 10 microsatellite markers. Plants coming from natural populations were grown in a common garden to evaluate the reproductive barriers between both species by means of controlled crosses. All the plants used for the field and greenhouse study were characterized by measuring traits related to plant size and flower size. We estimated the genetic molecular variances, the genetic differentiation, and the genetic structure by means of the F-statistic and Bayesian inference. We also estimated the amount of recent gene flow between populations. We found a narrow unimodal hybrid zone where the hybrid genotypes appear to have been maintained by significant levels of a unidirectional gene flow coming from parental populations and from weak reproductive isolation between them. Hybrid plants exhibited intermediate or vigorous phenotypes depending on the analyzed trait. The phenotypic differences between the hybrid and the parental plants were highly coherent between the field and controlled cross experiments and through time. The highly coherent results obtained by combining field, experimental, and genetic data demonstrate the existence of a stable and narrow unimodal hybrid zone between *Erysimum mediohispanicum* and *Erysimum nevadense* at the high elevation of the Sierra Nevada mountains.

## Introduction

Evaluating the mechanisms promoting the rise of hybrid zones helps us to understand the nature and dynamics of these interesting evolutionary scenarios. Hybrid zones have been pointed to as one of the most insightful places to study the evolutionary interactions between divergent but related taxa (Barton and Hewitt, 1985; Harrison, 1993). Important effects of hybrid zones were well documented on, for example, morphological traits (Tastard et al., 2012, Keller et al., 2021), behavior (Good et al., 2000), selective regimes (Cruz et al., 2001; Knief et al., 2019), and biodiversity (Whitham et al., 1994, 1999). The hybrids can show a modified phenotype promoting their evolvability and, by altering the reproductive barriers between the parental species, modify the evolvability of these lasts (Parsons et al., 2011). However, an integrative study of the mechanisms, patterns, and consequences of hybridization is not straightforward.

**Figure 1:**
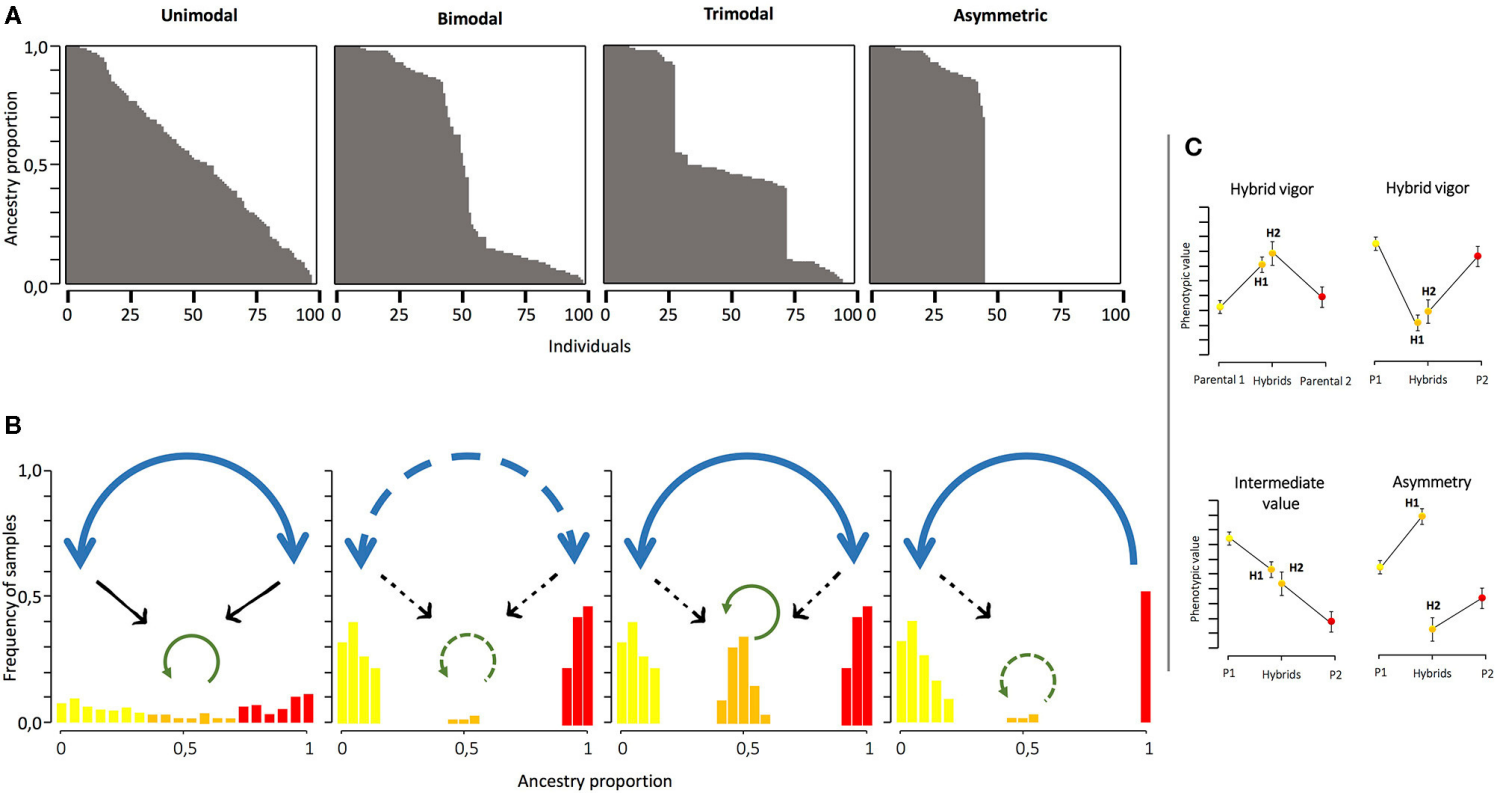
**(A)** Different models of hybrid zones based on the different patterns of the genetic mixture between divergent taxa. The abscissa axis shows individuals in the hybrid zone and the ordinate axis shows the ancestry proportion exhibited by individuals. **(B)** Frequency of each range of ancestry proportion in the population and the gene flow expected in each situation. Blue, green, and black arrows represent the gene flow promoted by interspecific crosses, backcrosses, and crosses between hybrids, respectively. The persistence of gene flow is indicated by solid lines; the absence of gene flow is indicated by broken lines. **(C)** Patterns of the phenotypic differentiation expected when differences between hybrids and parental plants are found. H1 and H2 are the hybrids obtained by adding pollen on parental plants 1 and 2, respectively. Yellow and red dots and bars correspond to the parental species, while orange represents hybrid values.

Even before the first conceptual models to explain hybrid zones were proposed, gene transfer between the neighboring species had been recognized as an important evolutionary process (Dobzhansky, 1940; Mayr, 1942; Anderson, 1949). The main classifications of hybrid zones proposed relate to the distribution of the hybrid and the parental phenotypes/genotypes (Harrison and Bogdanowicz, 1997; Jiggins and Mallet, 2000). Therefore, hybrid zones can be classified as unimodal, bimodal, trimodal, and asymmetric (Pickup et al., 2019). Unimodal hybrid zones are characterized by a higher frequency of individuals with intermediate genotypes between both parental species (Jiggins and Mallet, 2000). Hybridization and admixture are predominant in this kind of hybrid zones, so weak reproductive barriers are expected in them (Figure 1). A special case of the unimodal hybrid zones appears when the hybrid zone forms a single panmictic population exhibiting individuals with variable degrees of genetic similarity to the parental forms, termed as “hybrid swarms” (Harrison and Bogdanowicz, 1997; Jiggins and Mallet, 2000). Hybrid swarms could present individuals coming from first or second hybrid generations or from different levels of back-crossing. In a bimodal hybrid zone, individuals belonging to the parental species co-occur with a very low frequency of their hybrids (Cruzan and Arnold, 1993; McMillan et al., 1997; Jiggins and Mallet, 2000; Vedenina and Helversen, 2003). This kind of hybrid zone appears when strong reproductive isolation barriers exist between the species previously (Harrison and Bogdanowicz, 1997; Jiggins and Mallet, 2000; Coyne and Orr, 2004; Figure 1). However, when reproductive isolation between taxa is only partial, a stable hybrid zone would arise, generating a trimodal hybrid zone that depends, on the one hand, on the differences in fitness between the parentals and the hybrids and, on the other hand, on the migration rates from the parental zones (Key, 1968; Barton and Hewitt, 1985; Jiggins and Mallet, 2000; Figure 1). Finally, asymmetric hybrid zones occur when strong asymmetries in gene flow and barrier strength promote hybridization and introgression in one direction, such that gene flow occurs only from one species to another (Pickup et al., 2019; Figure 1).

Hybridization is much more frequent and is evolutionarily relevant in plants than in animals, but surprisingly, in the past, it has been more explored in the latter (Ellstrand et al., 1996; Dowling and Secor, 1997). The number of plant hybrid zones studied using an integrative approach, including genetic analyses and the nature of reproductive isolation, is limited (Abbott, 2017). However, interesting examples of contact zones and hybrid populations have been described in plants, for example, those described in *Phlox* (Levin, 1967), *Iris* (Cruzan and Arnold, 1993; Young, 1996; Emms and Arnold, 1997), *Helianthus* (Rieseberg et al., 1998), or *Armeria* (Fuertes Aguilar et al., 1999) and, more recently, those described in *Ipomopsis* (Campbell and Aldridge, 2006), *Silene* (Minder et al., 2007), *Narcissus* (Marques et al., 2010), or *Primula* (Keller et al., 2021), among others. These studies have demonstrated how the rise of hybrids can not only generate new phenotypic values in the contact zone but also modify the parental phenotype through introgression (Owens et al., 2016; Nieto Feliner et al., 2019) or by affecting the selective trajectories in the contact zone and the surrounding areas (Wielstra et al., 2017; Wielstra, 2019). Therefore, it is interesting to explore the phenotypic differences between the parental plants and hybrids not only in natural populations but also in controlled conditions where we could establish the origin and the level of admixture of the hybrid plants. In this sense, the hybrids can show different patterns of phenotypic differentiation. The hybrid phenotype could exhibit higher, lower, or intermediate phenotypic values compared with the parental plants (Figure 1C). The hybrid can also show an asymmetric pattern when the hybrids on one parental direction (that is, the parental direction acting as the mother) exhibit significant differences with the hybrid in the other sense (Figure 1C). Exploring this question through time in natural populations would allow us to know more about the stability of hybrids and the hybrid zones. However, the longer the life cycle of the organism being experimented on, the more difficult exploring becomes.

Incomplete reproductive barriers (or absence of them) are necessary to the occurrence of hybrids in a contact zone between different species. So, the evaluation of the strength of reproductive barrier components between the hybridizing species sheds light on the mechanisms underlying the rise of hybrid zones. Again, this objective is not always easily reachable, especially when the study systems demand several to many years for blooming. Reproductive barriers are usually estimated by the use of one or a few components of fitness. However, quantifying the different fitness components through the entire life cycle of plants will allow for a more realistic estimation of the intensity of these barriers and their cumulative effects on gene flow between the parental species and hybrids (Baack et al., 2015).

In this study, we examined two plant species belonging to the genus *Erysimum* L., *Erysimum mediohispanicum*, and *Erysimum nevadense*, and the hybrids produced between them in the Sierra Nevada (Southeast Spain). Genus *Erysimum* L. (Brassicaceae) is mainly distributed in the northern hemisphere (Polatschek, 1986), presenting an important diversification center in the western Mediterranean region (Greuter et al., 1986). The genus presents a complex evolutionary history due to events of interspecific hybridization and polyploidization, producing a highly diversified genus (more than 200 species) enriched in species complexes and cryptic species (Clot, 1992; Ancev, 2006; Marhold and Lihová, 2006; Turner, 2006; Couvreur et al., 2010, Abdelaziz et al., 2011; Al-Shebaz, 2012). In the Sierra Nevada, *E. mediohispanicum* and *E. nevadense* show contrasting distributions, inhabiting the lowland and the top of the mountains, respectively (Blanca et al., 2009). However, these species make what could be a secondary contact (Abdelaziz et al., 2014) at mid-elevation.

In the present study, we used an integrative approach by combining fieldwork, lab experiments, and molecular data to evaluate the nature and the strength of barriers to hybridization, to characterize the hybrid zone between *E. mediohispanicum* and *E. nevadense* in the Sierra Nevada National Park, and to explore the consequences of this geographic contact on plant phenotypes. The main goals of this study are (1) to explore the genetic and phenotypic differences between both species and their hybrids in the contact zone; (2) to explore the gene flow patterns promoting hybridization in this area; (3) to analyze the stability of the contact zone through time; and (4) to analyze the reproductive barriers promoting or constraining the rise of hybrids between *E. mediohispanicum* and *E. nevadense*.

## Materials and Methods

### Study System

*Erysimum mediohispanicum* Polatschek is an endemic species of the Iberian Peninsula, where it is distributed in two widespread and almost disconnected regions, located in the North and Southeast of the peninsula. Its life cycle varies among individuals and populations, usually being monocarpic. In the Sierra Nevada (Southeast of Spain), *E. mediohispanicum* is composed of diploid populations (Muñoz-Pajares et al., 2018) of facultative biennial, spending 2–3 years growing at an elevation of up to 2,200 m like a vegetative rosette on calcareous soils (Muñoz-Pajares et al., 2020). After that period, plants display flowers on one to three long stalks, ranging in number from only a few to several hundred (Gómez, 2003). These flowers are visited by a highly diverse assemblage of insects (Gómez et al., 2007; Gomez et al., 2014).

*Erysimum nevadense* Reut. is also a diploid species (author's unpublished data) and mostly polycarpic herb endemic to the peaks of the Sierra Nevada mountains, where it grows on siliceous soils at an elevation from 2,300 to 2,700-m (Blanca et al., 2009). *E. nevadense* spends 2–3 years like a rosette before displaying anything from a few to several hundred flowers on various short stalks. It is also a pollination-generalist plant, but it does not present a pollinator assemblage as diverse as that of *E. mediohispanicum*, probably due to the harsh conditions of its habitat (Gómez et al., 2007; Mohamed, 2013).

Both species come into contact along a narrow area at an elevation of approximately 2,200 m. In this contact zone, populations from each species are located at a mere hundred meters apart, generating an area where the phenotypic traits defining each species are difficult to identify. In 2007, we established a line transect of 3.1 km between these species in the north face of the Sierra Nevada encompassing five populations (Table 1), two *E. mediohispanicum* populations (Em25 and Em17), two *E. nevadense* populations (En11 and En10), and a putative hybrid population (H01), where both species contact. After 10 years, in 2017, we phenotypically resampled the hybrid population (H01) and the two parental populations more genetically related to the hybrid one (Em25 and En10).

**Table 1:** Geographic information and sampling effort in the five populations included in the study (ordered by elevation).

**Population characteristics**				**Sampling effort**				
				**2007**		**2009–2010**		**2017**
**Population**	**Latitude**	**Longitude**	**Elevation**	**Phenotyped plants**	**Genotyped plants**	**Transplanted plants**	**Crossed plants**	**Phenotyped plants**
Em25	37°7.230' N	3° 26.082' W	2,064	90	30	30	7	90
Em17	37° 6.698' N	3° 25.450' W	2,182	90	30	30	23	–
H01	37°6.908' N	3° 25.250' W	2,200	90	80	–	–	90
En11	37°6.750' N	3° 25.048' W	2,222	90	27	30	10	–
En10	37°6.658' N	3° 24.301' W	2,322	90	30	30	4	90

### Plant Phenotyping

For about a month from the end of May in 2007, at each of these populations, we phenotyped 90 plants by measuring the following phenotypic traits: (1) number of flowers and (2) number of stalks: counting the total number of flowers and stalks produced by each plant, respectively; (3) stalk height: the height of the tallest stalk of the plant from the ground to the top of the stalk at the end of the flowering period, when no more flowers are expected on the stalk, using a measuring tape (±0.5 mm error); (4) stalk diameter: the basal diameter of the tallest stalk; (5) corolla diameter: the distance between the edge of the two opposite petals; (6) corolla tube width: the diameter of the corolla tube aperture as the distance between the bases of two opposite petals; and (7) corolla tube length: the distance between the corolla tube aperture and the base of the sepals. The stalk diameter and the traits related to the flower size (4–7, above) were quantified in millimeters using a digital caliper (±0.01 mm. error). On approximately the same dates in 2017, all these phenotypic traits were remeasured in the same number of plants in the populations Em25, H01, and En10 (Table 1).

For each population and sampled year, we calculated the product–moment correlations, the covariances between each couple of the phenotyped traits, and the variance per trait (Supplementary Tables 1, 2). Kruskall–Wallis tests and one-way ANOVAs were used to compare the trait distributions and means of the populations for each phenotypic character, respectively. These analyses were performed using the package stats in R (R Development Core Team, 2014).

### DNA Isolation and Genotyping

In 2007, we collected fresh tissues from all the phenotyped plants at the putative hybrid area and also from randomly selected 30 plants out of the 90 phenotyped plants from each of the four parental populations in the transect (in total, 210 plants). The tissue was stored in silica gel for the subsequent DNA isolation, using the GenElute Plant Genomic DNA Miniprep kit (Sigma-Aldrich, St. Louis, MO, USA). The isolated DNA was used for the individual genotyping, carried out with 10 microsatellite markers previously described by Muñoz-Pajares et al. (2011). We performed PCR in a 15-μL reaction mixture containing 0.17 ng/μL of the template genomic DNA, 1X buffer (ref. M0273S, New England BioLabs), 0.16 mM each of dNTP (Sigma-Aldrich), 0.33 μM each of forward (fluorescently tagged) and reverse primers, and 0.02 U/μL *Taq* polymerase (ref. M0273S, New England Biolabs). PCR was conducted in a Gradient Master Cycler Pro S (Eppendorf, Hamburg, Germany) with an initial step of 30 s of denaturation at 94°C followed by 35 cycles at 94°C for 15 s, annealing temperatures for each microsatellite marker described in Muñoz-Pajares et al. (2011) for 30 s, extension at 72°C for 30 s, and a final extension step at 72°C for 3 min. PCR products were diluted at a ratio of 1:15 and sent to MACROGEN (Geumchun-gu, Seoul, South Korea; http://www.macrogen.com) for capillary electrophoresis using 400HD ROX as standard. Alleles were called using the Peak Scanner Software version 1.0 (Applied Biosystems).

### Genetic Data Analyses

Once we established the multilocus genotypes, we excluded the individuals presenting levels of missing data higher than 30% from the genetic analyses. This included 3 individuals from the En11 population and 10 individuals from H01 (Table 2). To genetically characterize the populations, we estimated the following parameters: (a) number of non-redundant multilocus genotypes (N_G_), as the number of genotypes showing at least one different allele, excluding missing data; (b) mean number of alleles per locus (n_a_); (c) observed heterozygosity (Ho), as the actual frequency of heterozygous individuals in the sample. We estimated the mean of the individual heterozygosities per population using the ratio between the number of heterozygote loci and the number of successfully genotyped loci; (d) gene diversity (Hs), as the expected proportion of heterozygous individuals assuming the Hardy–Weinberg equilibrium. Gene diversity was calculated using the Nei (1987) estimator; (e) mean allelic richness per locus (Rs), estimated as the probability of sampling the allele i at least once among the 2n genes of a sample, being independent of sample size; (f) the mean private allelic richness (R_P_), estimated as the mean number of singular alleles per locus presented at each population. All the previous parameters were calculated using the package hierfstat v. 0.04–6 (Goudet, 2005) or using scripts developed by ourselves, both in R (R Development Core Team, 2014). (g) Inbreeding coefficient (F_IS_), which provides information about the Hardy–Weinberg equilibrium departures due to either excess or defect of heterozygotes. We estimated F_IS_ by Bayesian inference using BayesAss v3.0 (Wilson and Rannala, 2003) for each population and overall for the two studied species. Analysis lasted for 10 million MCMC iterations, sampling every 1,000 generations and optimizing the mixing parameter for allele frequencies and for inbreeding coefficients. After that, we removed the first 10% of total iterations and checked the trace files with the program Tracer v1.4 (Rambaut and Drummond, 2007) to determine the convergence of the independent Bayesian MCMC runs.

**Table 2:** Genetic diversity parameters and effective population sizes for *Erysimum mediohispanicum* (Em), E*rysimum nevadense* (En), and the hybrid populations (total *N* = 197).

**Population**	***N***	***N_***G***_***	***n_***a***_***	***H_***O***_***	***H_***S***_***	***R_***S***_***	***R_***P***_***	***F_***IS***_***
Em25	30	30	7.8 ± 1.31	0.63 ± 0.04	0.70 ± 0.06	7.39 ± 1.30	0.50 ± 0.22	0.363 ± 0.248
Em17	30	30	7 ± 1.12	0.54 ± 0.05	0.67 ± 0.07	6.58 ± 0.97	0.10 ± 0.10	0.213 ± 0.045
H01	80	80	9.9 ± 1.57	0.61 ± 0.05	0.70 ± 0.06	7.54 ± 1.12	1.20 ± 0.36	0.139 ± 0.019
En11	27	26	6 ± 1.16	0.50 ± 0.07	0.64 ± 0.06	5.87 ± 1.09	0.20 ± 0.13	0.298 ± 0.068
En10	30	30	7.8 ± 1.57	0.57 ± 0.07	0.68 ± 0.06	7.06 ± 1.34	0.70 ± 0.33	0.360 ± 0.249
Total Em	60	60	7.4 ± 0.85	0.59 ± 0.03	0.68 ± 0.04	6.98 ± 0.79	0.30 ± 0.13	–
Total En	57	56	6.9 ± 0.97	0.53 ± 0.05	0.66 ± 0.04	6.47 ± 0.85	0.45 ± 0.18	–

*For each population, the parameters include the number of genotyped plants (N) with 10 nuclear microsatellite loci, the number of multilocus genotypes (N_G_), the mean number of alleles per locus (n_a_), the mean observed heterozygosity (H_O_), the mean gene diversity (H_S_), the mean allelic richness (R_S_), the mean private allelic richness (R_P_), and inbreeding coefficient (F_IS_). ±SD values are indicated. Mean (±SD) values for each population and species are also given*.

Microsatellite-based genetic differentiation among groups of populations belonging to the same species, among populations within groups, and among individuals within populations was estimated using a hierarchical analysis of molecular variance (AMOVA), as implemented in Arlequin (Excoffier and Lischer, 2010), using 1,000 permutations to test the significance. We performed this analysis two times: the first one excluding the hybrid population and the second one including the hybrid population in its own group. In addition, pairwise comparisons for genetic differentiation between populations were computed using the package hierfstat in R (R Development Core Team, 2014) by the computation of FST statistics (Weir and Cockerham, 1984) and *D*_*ST*_ (Jost, 2008). *F*_*ST*_ significance was calculated from 1,000 permutations, while the DST was estimated as the harmonic mean across loci.

The genetic relationship among genotypes was inferred by Bayesian means using the model-based clustering algorithm, as implemented in Structure v.2.2 (Pritchard et al., 2000; Falush et al., 2003). The number of multilocus genotype clusters (K) was analyzed using diploid setting and using admixture and *prior information* as ancestry models and correlations as the allele frequency model using prior information. We performed simulations with 10 replicates for each K value, ranging from K = 1 to K = 6. Each run consisted of 50,000 Markov Chain Monte Carlo (MCMC) steps after 20,000 burn-in steps. To detect the optimum value of K, we applied the Evanno method (Evanno et al., 2005), as implemented in the Structure Harvester website (Earl and von Holdt, 2012).

Finally, we estimated the gene flow rates among the studied populations and their significance by means of Bayesian inference using BayesAss v3.0 (Wilson and Rannala, 2003). Analysis lasted for 10 million MCMC iterations, with a sampling frequency of every 1,000 generations, optimizing the mixing parameter for allele frequencies and for inbreeding coefficients. After that, we removed the first 10% of the total iterations and checked the trace files with the program Tracer v1.4 (Rambaut and Drummond, 2007) to determine the convergence of the independent Bayesian MCMC runs.

### Reproductive Barrier Evaluation

In September 2009, we collected 120 individual plants from the four parental populations (30 plants per population of *E. mediohispanicum* and *E. nevadense*; Table 1). These individuals were transplanted to individual pots (11 cm x 11 cm x 15 cm) using the same soil in which they were growing and moved to a common garden in the University of Granada (an elevation of ~700-m). By May 2010, 44 individuals had survived (30 *E. mediohispanicum* plants and 14 *E. nevadense* plants; Table 1). Before the plants started blooming, they were moved to a greenhouse to exclude them from pollinators. During flowering, they were phenotyped following the abovementioned methodology used for the field plants, and several additional flowers per individual were subjected to two different treatments: (a) outcrossing (OC), in which some flowers were emasculated before opening and were pollinated with pollens from a different conspecific individual; and (b) interspecific hybridization (HC), in which some flowers were emasculated before opening and were pollinated with pollens from an individual from the other species. In total, 232 flowers were used in the experiment, with a mean of 5.5 ± 3 flowers per plant (3.2 ± 1.9 for OC and 2.8 ± 1.7 for HC per plant).

Once the blooming period was over, we recorded the number of flowers per plant and the treatment setting as ripe fruits or aborting without producing any fruits. The total number of ovules, unfertilized ovules, aborted seeds, and ripe seeds produced per ripe fruit were recorded in the lab using magnifying glasses. A total of 3,139 seeds were harvested at the end of the experiment. Subsequently, when possible, 15 seeds per plant and treatment were taken at random and sown randomly in a greenhouse. Their germination was recorded two times a week for the first month, and their survival was recorded every month during the next 10 months.

The following predispersal and postdispersal components of the plant reproductive output were quantified for each treatment and plant: (a) fruit set, the proportion of labeled flowers setting fruit; (b) seed production, the number of seeds produced per ovule in a given fruit; (c) seedling emergence, the proportion of sown seeds germinating and emerging as seedlings; and (d) seedling survival, the proportion of seedlings surviving until the end of the experiment. Afterward, we calculated the cumulative pre-dispersal fitness (*W*_*PRE*_), as fruit set x seed production, and the cumulative total fitness (*W*_*TOT*_), as fruit set x seed production x seedling emergence x seedling survival. The F1 generation resulting from OC and HC was grown until blooming (in the spring of 2011) when it was also phenotyped as we did with the natural populations.

Hybrid inviability (HI) was calculated per population by comparing the fitness between the intraspecific and interspecific crosses using the Ågren and Schemske (1993) approach as

(1) $$\begin{array}{l} HI=1-w_{\text{h}}/w_{\text{o}};w_{\text{h}}<w_{\text{o}} \end{array}$$

(2) $$\begin{array}{l} HI=w_{\text{h}}/w_{\text{o}}-1;w_{\text{h}}>w_{\text{o}} \end{array}$$

where w_o_ is the fitness of the intraspecific outcrossing treatments and w_h_ is the fitness of the hybrid crosses. In all cases, we used both predispersal and total fitness. Using this approach, the values range between −1 and +1. Positive values indicate that the hybrid crosses have lower fitness than intraspecific crosses (occurrence of hybrid inviability and hence the rise of reproductive barriers). The significant values of HI were calculated by computing 95% CIs by means of bootstrapping with 1,000 permutations, using package boot in R (Canty and Ripley, 2017).

## Results

### Phenotypic Differentiation

**Figure 2:**
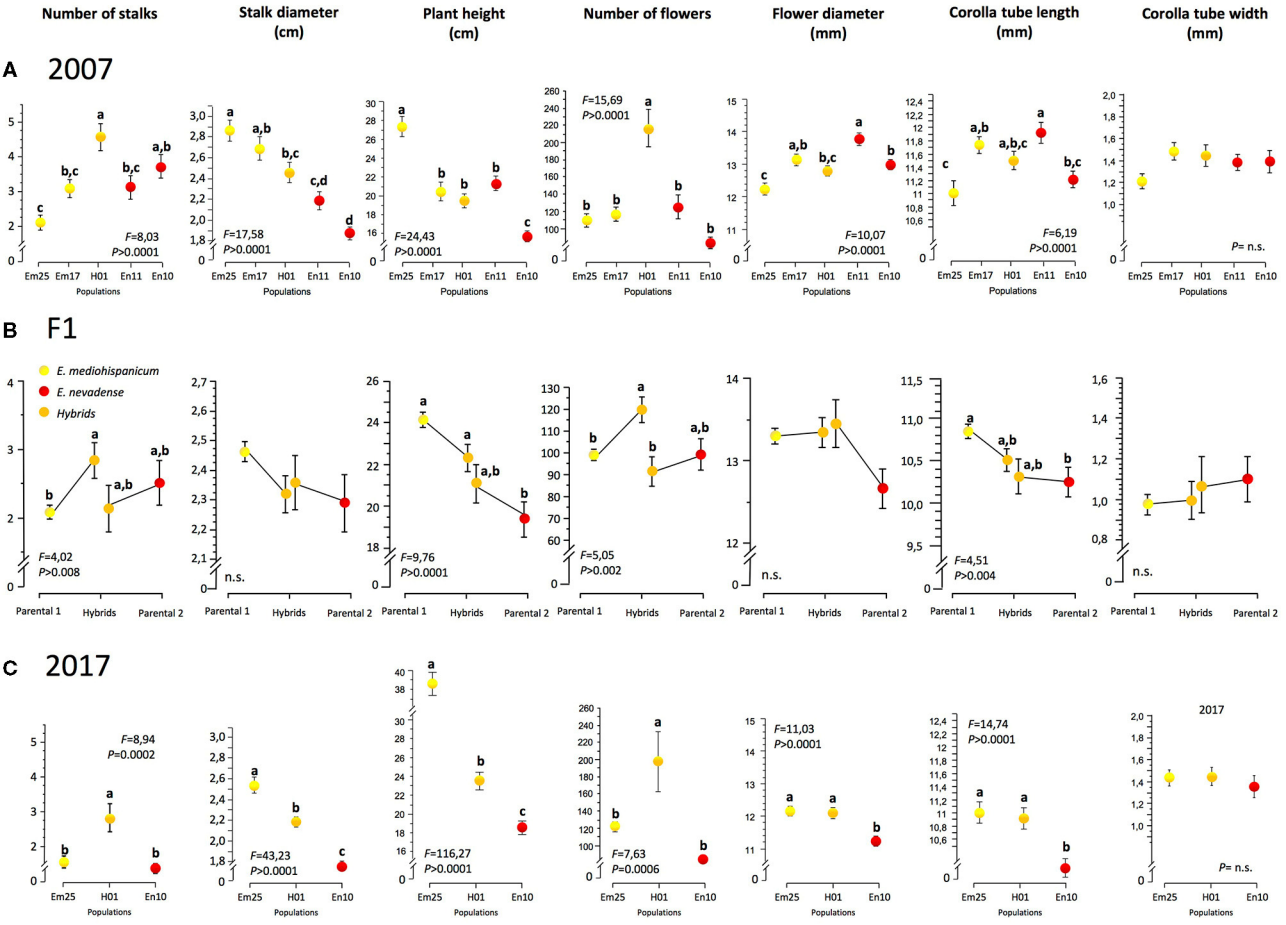
Values of phenotypic traits at each population in 2007 **(A)**, 2017 **(C)**, and the hybrid and parental plants obtained by controlled cross experiments **(B)**. Yellow dots denote plants or populations of *Erysimum mediohispanicum*, orange dots denote the hybrid plants or population, and red dots denote plants or populations of *Erysimum nevadense*. F-ratios refer to one-way ANOVAs. Letters indicate the groups of plants where the differences are significant, according to Tukey's HSD comparison.

Using one-way ANOVAs, we found significant differences at the among-population level in the 2007 sample for all the measured phenotypic traits, except for the corolla tube width (Figure 2A). The hybrid population showed the highest values for the number of flowering stalks and the number of flowers, both traits related to plant size. However, it showed intermediate values for the rest of the traits, including characters related to plant size (stalk diameter and plant height) and to flower size (Figure 2A). The same pattern was found again when we resampled three out of the original populations in 2017 (Em25, H01, and En10; Table 1 and Figure 2C).

The analysis of phenotypic traits performed on the offspring (F1 generation) of the controlled crosses confirmed these trends but also showed different patterns depending on the direction of gene flow producing the hybrid plant. The plants resulting from the hybrid crosses performed on *E. mediohispanicum*, i.e., *E. mediohispanicum* acting as the female, showed significantly higher values than the parental species for the number of flowering stalks and the number of flowers, while the hybrid plants resulting from crosses on *E. nevadense* showed lower values for these same traits (Figure 2B). For the rest of the phenotypic traits, the hybrid plants presented intermediate values, not significantly different from the values shown by the parentals for stalk diameter, flower diameter, and flower tube width (Figure 2B).

We found significant correlations in every population between the phenotypic traits defining the plant size (number of stalks, stalk diameter, plant height, and the number of flowers) and between the phenotypic traits defining the flower size (corolla diameter, corolla tube length, and corolla tube width) (Supplementary Table 1). In some cases, we also found significant positive correlations between the plant-size traits and the flower-size traits. This means that larger plants can also produce larger flowers, but larger plants do not present smaller flowers in any case. This pattern was found for the plants measured in the natural populations in 2007 (Supplementary Table 1) and 2017 (Supplementary Table 2), as well as in the plants obtained by the controlled crosses. The plants obtained by the intraspecific crosses presented similar number and levels of correlations to the plants phenotyped in natural populations. The experimental within-species cross plants showed similar patterns to those found in plants produced from hybrid crosses, but *E. nevadense* hybrids, where the number of traits significantly correlated, decreased (Supplementary Table 3).

### Genetic Diversity and Differentiation

A total of 197 plants were successfully genotyped from the five sampled populations in 2007 (Table 2). A total of 196 multilocus genotypes were identified from them, finding no identical multilocus genotypes among populations, but only one repeated multilocus genotype at population En11 (Table 2).

The mean number of alleles per locus (*n*_*a*_) ranged between 6.0 and 9.9, corresponding to En11 and H01, respectively, and *E. mediohispanicum* presented slightly higher values than *E. nevadense* (Table 2). The observed *H*_*O*_ presented values from 0.50 to 0.63, corresponding to these extremes to En11 and Em25, respectively, and again *E. mediohispanicum* values being higher. Moreover, the mean *H*_*S*_ presented the same pattern shown by *H*_*O*_ but the values of *H*_*S*_ were higher than *Ho* in all the considered populations and species (Table 2). The values of the mean allelic richness (*R*_*S*_) ranged from the minimum value (5.87) at En11 to the maximum (7.54) at H01, presenting higher values for *E. mediohispanicum* populations (Table 2). The pattern exhibited by the mean private allelic richness (*R*_*P*_) was different, being higher for *E. nevadense* and presenting their minimum value at Em17 (0.10) and the maximum value at H01 (1.20). Finally, the inbreeding coefficient (*F*_*IS*_) exhibited its minimum value at the hybrid population (0.139) and the maximum values at both extremes of the transect (Em25 = 0.363 and En10 = 0.360), although the SEs associated with these maximum values were also higher (Table 2).

Hierarchical analysis of molecular variance (AMOVA) indicated that population groups (species) were not genetically differentiated for these markers (Table 3). Moreover, this also occurred when H01 was included as a separate group. In contrast, the among-population-within–groups level (both including and excluding H01 in the analysis) was significantly differentiated, although it accounted for only low amounts of genetic variance (Table 3). The genetic variance at this level decreased when the hybrid population was included (Table 3). The within-population level accounted for almost all genetic variance in both cases (Table 3).

**Table 3:** Hierarchical analysis of molecular variance (AMOVA).

	**A: [Em25–Em17][En11–En10]**				**B: [Em25–Em17]–H01-[En11–En10]**			
**Source of variation**	***d.f.***	**Variation (%)**	**Fixation index**	***P*** **-Value**	***d.f.***	**Variation (%)**	**Fixation index**	***P*** **-Value**
Among groups	1	0.000	0.000	0.69306	2	0.000	0.000	0.93939
Among populations within groups	2	2.31	0.023	0.00489	2	1.48	0.015	0.00782
Within populations	230	97.69	0.034	0.00391	389	98.52	0.033	0.00293
Total	233				393			

*(A) Two groups (E. mediohispanicum; E. nevadense) were established, excluding the H01 population. (B) Three groups were established (E. mediohispanicum populations; E. nevadense populations; hybrid population). The degrees of freedom (d.f.), percentage of variation in each hierarchical level, and their respective fixation indexes with their p-values are given*.

Pairwise *F*_*ST*_ comparisons between populations showed that values ranging from a maximum differentiation of 0.0368 between Em17 and En10 to a minimum differentiation value of 0.0107 exhibited between Em25 and H01 (Table 4). Despite the low pairwise *F*_*ST*_ values found in our transect, all of them were significant after Bonferroni correction (Table 4). In addition, pairwise *D*_*ST*_ comparisons between populations indicated a similar pattern of genetic differentiation, with the higher *D*_*ST*_ value between Em25 and Em17 and between Em17 and En10 and the lower one between Em25 and H01 (Table 4).

**Table 4:** Pairwise *F*_*ST*_ (above diagonal) and *D*_*ST*_ (below diagonal) values along the transect, estimated by means of Weir and Cockerham methods 1984 and as the harmonic mean across loci, respectively (Weir and Cockerham, 1984).

	**Em25**	**Em17**	**H01**	**En11**	**En10**
Em25		0.0321^**^	0.0107^*^	0.0250^*^	0.0325^**^
Em17	0.055		0.0228^**^	0.0265^**^	0.0368^**^
H01	0.013	0.038		0.0179^**^	0.0128^**^
En11	0.043	0.042	0.032		0.0267^**^
En10	0.032	0.054	0.014	0.037	

*The significance for F_ST_ values was calculated from 1,000 permutations and the p-values resulted after the Bonferroni correction*:

* *P < 0.05*,

** *P < 0.01*.

### Genetic Structure and Gene Flow

**Figure 3:**
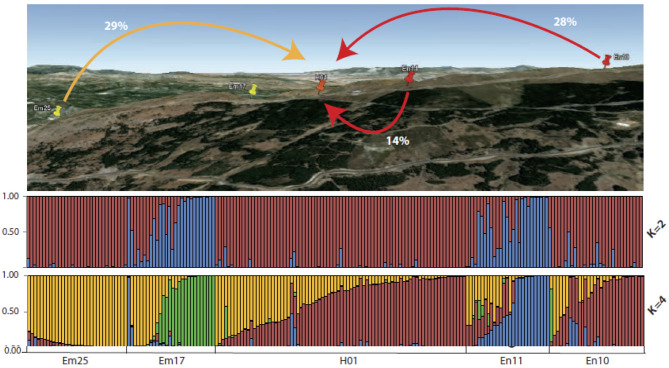
Significant gene flow values among *E. mediohispanicum* and *E. nevadense* populations surrounding the hybrid zone. Bottom: diagram with individual barplots from STRUCTURE (K = 2 and K = 4) based on the variation of 10 unlinked microsatellite loci.

The Bayesian inference of the genetic structure assigned the studied populations to two genetic clusters (K = 2), the second-most probable model being the one considering four genetic clusters (K = 4) (Supplementary Table 4). Considering K = 2, the plants belonging to the populations Em25, En10, and H01 exhibited very high membership proportions to a given ancestral genetic cluster, while the plants belonging to Em17 and En11 showed higher or medium values of membership proportions to the second cluster (Figure 3). However, when we consider the model assuming K = 4, the plants from *E. mediohispanicum* and *E. nevadense* populations showed medium to high membership proportions to four ancestral genetic clusters (Figure 3). In contrast, the individuals from the hybrid population exhibited medium to high values for their assignment probabilities to the most frequent genetic clusters in Em25 and En10 (Figure 3).

The Bayesian inference indicated that the highest gene flow levels were taking place at the intrapopulational level for all the populations included in the study (Table 5). The gene flow among populations was not significant, except for the gene flow from Em25, En11, and En10 to the hybrid population (Table 5), the gene flow being higher from Em25 (29%) and En10 (28%) to H01 than that from En11 (14%).

**Table 5:** Recent migration rates (*m*_*ij*_) and SEs from *i* populations to *j* populations in the transect between *E. mediohispanicum* and *E. nevadense*, inferred from the variation at 10 microsatellite DNA loci using BayesAss.

		**Population** ***j***				
		**Em25**	**Em17**	**H01**	**En11**	**En10**
	Em25	**0.677 ± 0.019**	0.010 ± 0.019	**0.287 ± 0.042**	0.018 ± 0.028	0.008 ± 0.016
	Em17	0.011 ± 0.022	**0.879 ± 0.076**	0.055 ± 0.065	0.045 ± 0.053	0.010 ± 0.018
Pop *i*	H01	0.004 ± 0.009	0.017 ± 0.021	**0.947 ± 0.046**	0.027 ± 0.041	0.005 ± 0.009
	En11	0.011 ± 0.023	0.019 ± 0.034	**0.141 ± 0.099**	**0.818 ± 0.098**	0.011 ± 0.020
	En10	0.008 ± 0.017	0.017 ± 0.026	**0.279 ± 0.047**	0.017 ± 0.031	**0.677 ± 0.021**

*Significant gene flow rates are shown in bold*.

### Reproductive Barriers

Significant barriers to reproduction were found between *E. mediohispanicum* and *E. nevadense*, considering the population and species level. However, these barriers were asymmetric. The hybrid crosses performed on *E. mediohispanicum* plants presented a similar fitness to the intraspecific crosses performed in this species, considering both the cumulative predispersal component of the fitness and the total cumulative fitness (Table 6). When *E. nevadense* plants received the pollen of *E. mediohispanicum*, we found significant positive HI values at both the population and species levels, independent of the predispersal or cumulative fitness. Hence, hybrid crosses showed a mean decrease in fitness between 36% (considering *Wpre* in En11) and 56% (calculated for *Wtot* for En10). Considering the species level, the *E. nevadense* hybrids showed a mean decrease of 40% compared with the intraspecific crosses (Table 6).

**Table 6:** Reproductive barrier estimation between *E. mediohispanicum* and *E. nevadense*.

	**Hybrid inviability**						
**Species**	***N***	***W***_***PRE***_	***W***_***TOT***_	**Pops**	***N***	***W***_***PRE***_	***W***_***TOT***_
*E. mediohispanicum*	30	0.00 [−0.22, 0.24]	0.12 [−0.16,0.45]				
				Em 25	7	0.10 [−0.5657, 0.9557]	0.38 [−0.2355, 1.4564]
				Em 17	23	−0.01 [−0.2025, 0.2196]	0.02 [−0.2210, 0.3582]
*E. nevadense*	14	**0.40 [0.18, 0.78]**	**0.41 [0.07, 1.31]**				
				En 11	10	**0.36 [0.1709, 0.8533]**	**0.45 [0.1408, 1.1338]**
				En 10	4	**0.53 [0.2189, 0.7835]**	**0.56 [0.3287, 0.7865]**

*The number of plants used in the control-crossing experiment (N) and hybrid inviability (comparing intraspecific and interspecific crosses) at predispersal (W_PRE_) and total (W_TOT_) fitness calculated for the hybrid crosses made on E. mediohispanicum and E. nevadense plants. We show the hybrid inviability values per population and per species and their 95% CI. Significant values are stressed in bold*.

## Discussion

The consequences of new contacts between the closely related species have been widely highlighted for their interest in understanding the evolutionary processes (Barton and Hewitt, 1985; Harrison, 1993). More recently, they have been pointed out for their importance in assisting the adoption of better conservation policies in the face of rising hybridization (Gómez et al., 2015). In this sense, *E. mediohispanicum* and *E. nevadense* were suggested to be the two non-sister species (Abdelaziz et al., 2014) with contrasting distribution and a karyotypic history in the Iberian Peninsula (Muñoz-Pajares et al., 2017, 2018). However, as in the Sierra Nevada National Park, *E. mediohispanicum* inhabits the low lands, while *E. nevadense* has been described as an endemic species of the Sierra Nevada peaks (Blanca et al., 2009); both species could be the result of a recent or ongoing process of evolutionary divergence by the adaptation to high mountains. This elevational parapatric distribution could promote divergent ecological selection patterns, as those observed in other plant species (Campbell, 2004; Muir et al., 2013), preventing gene flow into the parental areas but creating a stable hybrid zone at the contact. This is supported in our case by the phenotypic stability of the contact zone after 10 years, and it is especially relevant because it makes the scenarios of non-sister species and non-completed speciation compatible as the origin of this hybrid zone. These hybridizing contact zones have important effects on the evolutionary dynamics of the hybridizing species and the biodiversity in the area where the contact happens (Wielstra, 2019; Alves de Moura et al., 2020). The hybridization is not always evident for human, which could have even more pervasive consequences for the biology of the species and their conservation (Gómez et al., 2015; Waldron et al., 2019).

With almost 200 genotyped plants along a transect from *E. mediohispanicum* to *E. nevadense*, we found significant differences between the populations, except for the putative hybrid population. This population seemed to be a mixture between genotypes coming from Em25 and En10, the populations in the extremes of the transect. Most of the genotypes of the hybrid population showed variable admixture proportions with the main genetic clusters of populations Em25 and En10. However, there were also plant genotypes belonging to one of the two inferred genetic sources in Em25 and En10 (Figure 3). Therefore, this hybrid zone shows the typical pattern found in the unimodal hybrid zones, with the probable presence of parental plants and/or backcrosses among hybrids, the more frequent plants in the population. These unimodal hybrid zones are frequent when the hybridizing species have not developed strong reproductive barriers (Harrison and Bogdanowicz, 1997). However, Jiggins and Mallet (2000) proposed that the unimodal and bimodal hybrid zones represent different stages of the speciation process: the former would correspond to the early stages in the speciation continuum, while the latter would signify that speciation is nearly completed (Mallet and Dasmahapatra, 2012).

The absence of an interspecific genetic structure and differentiation found in the altitudinal transect from *E. mediohispanicum* to *E. nevadense* seems to be related to the significant and unidirectional levels of gene flow detected for the hybrid population. These patterns are probably related to the highly diverse pollinator assemblage interacting with these species in the Sierra Nevada (Gómez et al., 2009a,b; Abdelaziz et al., unpublished data) and to their ability to promote pollen movement between populations (Tochigi et al., 2021; Abdelaziz et al., unpublished data), as supported by the high number of multilocus genotypes identified in this study. We could identify the hybrid zone despite the nearby distribution of our populations in this area. Usually, the hybrid zones have been studied in extended areas where the researchers identify parental populations and hybrids in a latitudinal gradient (e.g., Lepais et al., 2009; Liu et al., 2014). However, when searching for hybrid contact zones on altitudinal gradients, the approach must be more local: first, because the area at the mountain peaks is reduced with the elevation, and second, because the changes of kilometers in latitude correspond to the changes of meters in elevation for the environmental conditions affecting the plants (Jump et al., 2009). The interest for studying hybrid zones in elevation gradients is increasing in recent years (Aizawa and Iwaizumi, 2020; Mimura and Suga, 2020; Tamaki and Yamada, 2020), although some of these hybrid zones are already in the central body knowledge of hybridization in plants (Aguilar et al., 1999; James and Abbott, 2005).

The gene flow associated with the seed or pollen movements is not enough to generate hybrids in a contact zone. Weak reproductive barriers between the contacting species are also needed, whatever the mechanisms or the way to estimate those (Baack et al., 2015). We calculated pre- and post-dispersal components of reproductive barriers between *E. mediohispanicum* and *E. nevadense*. All of them were coherent and found evidence of the existence of asymmetric reproductive barriers between both species. In this sense, the hybrids obtained by pollinating the plants of *E. nevadense* experienced significant reductions of fitness when compared with the intraspecific crosses at both the predispersal (seed production) and postdispersal (germination and survival) components. In contrast, the hybrid crosses performed on *E. mediohispanicum* showed no fitness differences with the intraspecific crosses. Moreover, the *E. mediohispanicum* hybrids displayed a hybrid vigor for such traits as the number of stalks and the number of flowers, while significantly reduced values for these traits were found for *E. nevadense* hybrids. This means that the *E. nevadense* hybrids suffer from an important reduction in fitness at the prezygotic and postzygotic levels. The traits found to be modified in hybrids are related to floral display and are far from irrelevant. Indeed, they were recurrently demonstrated to play an important role in the attraction of pollinators and being under significant natural selection regimes in *Erysimum* (Gómez et al., 2009a) and other plant species (e.g., Johnston, 1991; Parachnowitsch and Kessler, 2010; Trunschke et al., 2017). Hence, the hybrids of *E. mediohispanicum* probably play an important role in maintaining the hybrid population.

In nature, the plants in the hybrid zone showed vigor only for traits related to the plant size (number of stalks and flowers), but intermediate values were shown for the rest (including all the flower size traits). Reductions in fitness have been described for hybrids mainly associated with sterility and developmental problems (Bomblies and Weigel, 2007; Ouyang et al., 2010; Levin, 2012), promoting the rise of efficient reproductive barriers (Ispolatov and Doebeli, 2009). However, hybrid organisms are also more vigorous than either of the parentals due to heterosis (Huang et al., 2015) or transgressive segregation in the further hybrid generations (Rieseberg et al., 1996, 1999). However, we have found that, while some traits can show heterotic patterns, some other traits can show just intermediate values to both parents. Depending on the trait modified by the hybridization, adaptive trajectories can significantly change and the evolutionary path of the hybrid population can move closer to or further away from the parental species. This fact, together with the absence of the important differences in flower size, may help in the occurrence of hybrids and backcrosses in the natural populations, favoring the continuity or even the spread of the hybrid population. Our phenotypic analysis, 10 years after the first sampling, confirms the same phenotypic pattern found in the transect in the first year and also found in the artificial hybrids developed in the greenhouse. Therefore, we can say that the hybrids are still produced in this contact zone. Studies on the stability of hybrids beyond F1 and on the natural selection acting on the parental and hybrid plants would clarify the stability of this hybrid zone and its effects on the biodiversity of the Sierra Nevada high mountains.

Despite the amount of data included in the current study, we could neither identify the reason why most of the gene flow to the hybrid population is coming from the most distant parental populations nor identify the causes of the asymmetry in the hybrid fitness. On the one hand, the non-homogeneous pattern of gene flow found in our altitudinal gradient could be related to the erratic movements or long-distance flights of the pollinators, as those described for *Syzygium tierneyanum* and *Delphinium nuttallianum* (Hopper, 1980; Schulke and Waser, 2001). The composition of the pollinator assemblage has an important impact on the gene flow and the genetic structure pattern found on the plant species interacting with them (Brunet et al., 2019). On the other hand, the mechanism underlying the asymmetry in hybrid fitness could be related to asymmetries in the interaction between the pollen and the stigma. A similar interaction seems to play a central role in the asymmetric patterns of hybridization found in different plant species (Rahmé et al., 2009; Pickup et al., 2019). Moreover, the outbreeding depression (Barmentlo et al., 2018) promoted by the intense patterns of local adaptation to the high elevation of the mountain could significantly contribute to the asymmetrical hybridization pattern found in this study.

## Conclusions

The highly coherent results obtained by combining the field, the experimental, and the genetic data demonstrate that, in the altitudinal gradient between *E. mediohispanicum* and *E. nevadense* studied in the present work, there exists a narrow unimodal hybrid zone. The hybrid population appears to be maintained by high levels of the incoming gene flow from mainly two parental populations and by the weak and asymmetrical reproductive barriers between the parental species. *E. mediohispanicum* tolerates the interspecific pollen, but this does not occur with *E. nevadense*. This places *E. mediohispanicum* in a better situation for introgression events than E. nevadense. This pattern was replicated in the greenhouse, where *E. mediohispanicum* hybrids presented some heterotic traits, while *E. nevadense* showed reduced values for these same traits. Finally, a similar pattern was also found in the field when the same traits related to plant sizes showed heterotic values in the hybrid population compared with those in the parental populations. The heterotic phenotypes are probably more frequent in the hybrid zone because of their advantage in attracting pollinators and improving fitness since these traits were demonstrated to be under selection in *E. mediohispanicum* (Gómez et al., 2009a). Finding the same pattern after 10 years is evidence of the stability of this hybrid zone between *E. mediohispanicum* and *E. nevadense* at the top of the Sierra Nevada mountains.

## Data Availability Statement

The original contributions presented in the study are included in the article/Supplementary Material, further inquiries can be directed to the corresponding author/s.

## Author Contributions

MA, AJM-P, FP, and JMG conceived and designed the experiments. MA, AJM-P, MB, and AG-M performed the experiments. MA, AJM-P, FP, JMG, MB, and AG-M analyzed the data. MA, AJM-P, FP, and JMG drafted and modified the manuscript. All authors read and approved the manuscript.

## Conflict of Interest

The authors declare that the research was conducted in the absence of any commercial or financial relationships that could be construed as a potential conflict of interest.

## Publisher's Note

All claims expressed in this article are solely those of the authors and do not necessarily represent those of their affiliated organizations, or those of the publisher, the editors and the reviewers. Any product that may be evaluated in this article, or claim that may be made by its manufacturer, is not guaranteed or endorsed by the publisher.
