# Supplementary material for: Asymmetric Reproductive Barriers and Gene Flow Promote the Rise of a Stable Hybrid Zone in the Mediterranean High Mountain
Source: Front Plant Sci. 2021 Aug 25;12:687094. doi: 10.3389/fpls.2021.687094 (PMC8424041; doi:10.3389/fpls.2021.687094)
Supplement: Supplementary file 1 [file Data_Sheet_1.docx]

**Supplemental information**

**Table S1.** Phenotypic correlations between plant traits per population in the year 2007. For each studied population 90 plants were measured. Above diagonal are product-moment correlations, below diagonal are covariances, and in diagonal is the variance per trait. *P<0.05, **P<0.01, ***P<0.001, ****P<0.0001

| **Em25** | **Number**  **of Stalks** | **Stalk**  **diameter** | **Plant**  **height** | **Corolla**  **diameter** | **Corolla**  **tube length** | **Corolla**  **tube width** | **Number**  **of flowers** |
| --- | --- | --- | --- | --- | --- | --- | --- |
| **Number of Stalks** | 4.055 | -0.068 | -0.153 | -0.151 | -0.208* | -0.054 | **0.547****** |
| **Stalk diameter** | -0.130 | 0.906 | **0.436****** | 0.147 | 0.112 | 0.114 | **0.511****** |
| **Plant height** | -3.175 | 4.266 | 105.612 | **0.354***** | 0.210* | 0.053 | **0.410****** |
| **Corolla diameter** | -0.541 | 0.250 | 6.468 | 3.167 | **0.559****** | 0.182 | 0.043 |
| **Corolla tube length** | -0.746 | 0.189 | 3.843 | 1.770 | 3.160 | 0.012 | -0.075 |
| **Corolla tube width** | -0.072 | 0.071 | 0.362 | 0.214 | 0.014 | 0.436 | -0.073 |
| **Number of flowers** | 82.220 | 36.295 | 314.509 | 5.670 | -9.978 | -3.620 | 5577.986 |
| **Em17** | Number  of Stalks | Stalk  diameter | Plant  height | Corolla  diameter | Corolla  tube length | Corolla  tube width | Number  of flowers |
| **Number of Stalks** | 5.902 | -0.286** | -0.112 | 0.104 | 0.102 | 0.066 | **0.436****** |
| **Stalk diameter** | -0.741 | 1.136 | **0.346***** | **0.391***** | **0.370***** | 0.227* | **0.4078****** |
| **Plant height** | -2.533 | 3.445 | 87.366 | **0.355***** | 0.250* | 0.005 | 0.314** |
| **Corolla diameter** | 0.411 | 0.678 | 5.401 | 2.649 | **0.655****** | 0.157 | 0.198 |
| **Corolla tube length** | 0.303 | 0.484 | 2.861 | 1.307 | 1.503 | 0.113 | **0.349***** |
| **Corolla tube width** | 0.122 | 0.183 | 0.038 | 0.192 | 0.105 | 0.568 | 0.254* |
| **Number of flowers** | 82.827 | 33.945 | 229.731 | 25.125 | 33.409 | 14.944 | 6105.990 |
| **H01** | Number  of Stalks | Stalk  diameter | Plant  height | Corolla  diameter | Corolla  tube length | Corolla  tube width | Number  of flowers |
| **Number of Stalks** | 14.451 | -0.073 | 0.055 | 0.045 | -0.002 | 0.080 | **0.646****** |
| **Stalk diameter** | -0.261 | 0.888 | 0.326** | 0.180 | 0.204 | -0.061 | **0.404****** |
| **Plant height** | 1.498 | 2.181 | 50.509 | 0.246* | 0.303** | -0.083 | 0.328** |
| **Corolla diameter** | 0.257 | 0.254 | 2.624 | 2.245 | **0.608****** | **0.422****** | 0.133 |
| **Corolla tube length** | -0.009 | 0.252 | 2.824 | 1.194 | 1.720 | -0.014 | 0.067 |
| **Corolla tube width** | 0.280 | -0.053 | -0.545 | 0.584 | -0.017 | 0.852 | -0.030 |
| **Number of flowers** | 501.156 | 77.707 | 475.995 | 40.656 | 18.006 | -5.683 | 41655.549 |
| **En11** | Number  of Stalks | Stalk  diameter | Plant  height | Corolla  diameter | Corolla  tube length | Corolla  tube width | Number  of flowers |
| **Number of Stalks** | 10.077 | -0.151 | -0.096 | -0.124 | -0.244* | 0.212* | **0.470****** |
| **Stalk diameter** | -0.383 | 0.635 | **0.487****** | 0.286** | 0.335** | 0.001 | **0.473****** |
| **Plant height** | -2.167 | 2.751 | 50.253 | 0.181 | 0.199 | -0.063 | **0.445****** |
| **Corolla diameter** | -0.718 | 0.416 | 2.343 | 3.315 | **0.558****** | **0.344***** | 0.095 |
| **Corolla tube length** | -1.118 | 0.385 | 2.036 | 1.466 | 2.081 | 0.003 | 0.019 |
| **Corolla tube width** | 0.453 | 0.001 | -0.300 | 0.421 | 0.003 | 0.453 | 0.073 |
| **Number of flowers** | 197.182 | 49.812 | 416.985 | 22.948 | 3.728 | 6.516 | 17480.013 |
| **En10** | Number  of Stalks | Stalk  diameter | Plant  height | Corolla  diameter | Corolla  tube length | Corolla  tube width | Number  of flowers |
| **Number of Stalks** | 9.995 | -0.109 | -0.113 | 0.114 | -0.021 | 0.255* | **0.691****** |
| **Stalk diameter** | -0.205 | 0.351 | 0.060 | 0.100 | **0.436****** | 0.042 | 0.249* |
| **Plant height** | -2.059 | 0.204 | 32.944 | 0.200 | 0.228* | -0.098 | 0.084 |
| **Corolla diameter** | 0.561 | 0.092 | 1.777 | 2.404 | **0.501****** | 0.254* | 0.128 |
| **Corolla tube length** | -0.083 | 0.320 | 1.618 | 0.962 | 1.533 | 0.104 | 0.194 |
| **Corolla tube width** | 0.778 | 0.024 | -0.544 | 0.380 | 0.124 | 0.929 | 0.225* |
| **Number of flowers** | 134.966 | 9.113 | 29.634 | 12.244 | 14.823 | 13.382 | 3812.000 |

**Table S2.** Phenotypic correlations between plant traits per population in the year 2017. For each studied population 90 plants were measured. Above diagonal are product-moment correlations, below diagonal are covariances, and in diagonal is the variance per trait. *P<0.05, **P<0.01, ***P<0.001, ****P<0.0001

| **Em25** | Number  of Stalks | Stalk  diameter | Plant  height | Corolla  diameter | Corolla  tube length | Corolla  tube width | Number  of flowers |
| --- | --- | --- | --- | --- | --- | --- | --- |
| **Number of Stalks** | 2,399 | 0,039 | 0,052 | 0,011 | -0,055 | 0,052 | **0,529****** |
| **Stalk diameter** | 0,052 | 0,742 | **0,361***** | 0,199 | 0,129 | -0,019 | **0,526****** |
| **Plant height** | 0,941 | 3,643 | 137,284 | 0,076 | 0,114 | 0,048 | **0,402****** |
| **Corolla diameter** | 0,025 | 0,241 | 1,249 | 1,988 | **0,478****** | 0,113 | -0,018 |
| **Corolla tube length** | -0,126 | 0,166 | 1,996 | 1,004 | 2,221 | -0,193 | 0,009 |
| **Corolla tube width** | 0,054 | -0,011 | 0,382 | 0,107 | -0,194 | 0,454 | -0,007 |
| **Number of flowers** | 56,362 | 31,153 | 323,648 | -1,748 | 0,917 | -0,305 | 4731,993 |
| **H01** | Number  of Stalks | Stalk  diameter | Plant  height | Corolla  diameter | Corolla  tube length | Corolla  tube width | Number  of flowers |
| **Number of Stalks** | 12,943 | -0,026 | 0,150 | 0,069 | -0,098 | 0,181 | **0,583****** |
| **Stalk diameter** | -0,052 | 0,305 | 0,330** | 0,224* | 0,306** | 0,017 | 0,223* |
| **Plant height** | 4,947 | 1,674 | 84,424 | 0,129 | 0,103 | -0,018 | **0,377***** |
| **Corolla diameter** | 0,386 | 0,191 | 1,837 | 2,395 | 0,316** | 0,332** | 0,046 |
| **Corolla tube length** | -0,547 | 0,263 | 1,466 | 0,762 | 2,420 | 0,086 | 0,049 |
| **Corolla tube width** | 0,493 | 0,007 | -0,128 | 0,389 | 0,101 | 0,575 | 0,077 |
| **Number of flowers** | 879,144 | 51,620 | 1453,852 | 29,849 | 31,945 | 24,435 | 175647,130 |
| **En10** | Number  of Stalks | Stalk  diameter | Plant  height | Corolla  diameter | Corolla  tube length | Corolla  tube width | Number  of flowers |
| **Number of Stalks** | 1,613 | 0,048 | 0,181 | -0,127 | -0,081 | -0,064 | **0,702****** |
| **Stalk diameter** | 0,029 | 0,226 | **0,398***** | 0,114 | 0,119 | -0,021 | **0,372***** |
| **Plant height** | 1,607 | 1,324 | 48,922 | 0,192 | 0,192 | -0,119 | **0,400****** |
| **Corolla diameter** | -0,237 | 0,080 | 1,976 | 2,167 | **0,593****** | 0,189 | -0,069 |
| **Corolla tube length** | -0,134 | 0,073 | 1,745 | 1,131 | 1,679 | -0,169 | 0,009 |
| **Corolla tube width** | -0,077 | -0,009 | -0,796 | 0,265 | -0,209 | 0,907 | -0,207 |
| **Number of flowers** | 27,676 | 5,491 | 86,950 | -3,163 | 0,347 | -6,117 | 963,808 |

**Table S3.** Phenotypic correlations between plant traits per parental and hybrid plants obtained in the controlled crosses experiments. For each studied population 90 plants were measured. Above diagonal are product-moment correlations, below diagonal are covariances, and in diagonal is the variance per trait. *P<0.05, **P<0.01, ***P<0.001, ****P<0.0001

| ***E. mediohispanicum* hybrids** | **Number**  **of Stalks** | **Stalk**  **diameter** | **Plant**  **height** | **Corolla**  **diameter** | **Corolla**  **tube length** | **Corolla**  **tube width** | **Number**  **of flowers** |
| --- | --- | --- | --- | --- | --- | --- | --- |
| **Number of Stalks** | 4,760 | -0,104 | -0,250* | -0,265* | -0,323** | 0,208* | **0,682****** |
| **Stalk diameter** | -0,121 | 0,280 | **0,466****** | **0,361***** | **0,381***** | 0,039 | 0,354** |
| **Plant height** | -2,967 | 1,339 | 29,467 | 0,337** | **0,465****** | -0,013 | 0,115 |
| **Corolla diameter** | -0,902 | 0,299 | 2,857 | 2,442 | **0,536****** | 0,298* | -0,021 |
| **Corolla tube length** | -0,819 | 0,235 | 2,940 | 0,976 | 1,356 | -0,029 | -0,021 |
| **Corolla tube width** | 0,361 | 0,017 | -0,055 | 0,370 | -0,027 | 0,633 | 0,163 |
| **Number of flowers** | 72,543 | 9,126 | 30,558 | -1,635 | -1,174 | 6,313 | 2375,208 |

| ***E. mediohispanicum* parentals** | **Number**  **of Stalks** | **Stalk**  **diameter** | **Plant**  **height** | **Corolla**  **diameter** | **Corolla**  **tube length** | **Corolla**  **tube width** | **Number**  **of flowers** |
| --- | --- | --- | --- | --- | --- | --- | --- |
| **Number of Stalks** | 2,501 | **-0,220***** | -0,166* | -0,107 | -0,096 | -0,083 | **0,604****** |
| **Stalk diameter** | -0,170 | 0,241 | 0,212** | 0,056 | **0,257***** | -0,080 | 0,198** |
| **Plant height** | -1,416 | 0,561 | 29,000 | **0,284****** | **0,336****** | 0,117 | 0,150* |
| **Corolla diameter** | -0,240 | 0,039 | 2,174 | 2,015 | **0,497****** | **0,415****** | -0,100 |
| **Corolla tube length** | -0,183 | 0,151 | 2,170 | 0,846 | 1,439 | 0,044 | 0,009 |
| **Corolla tube width** | -0,102 | -0,030 | 0,486 | 0,454 | 0,040 | 0,594 | -0,105 |
| **Number of flowers** | 44,061 | 4,483 | 37,205 | -6,580 | 0,493 | -3,746 | 2128,288 |

| ***E. nevadense***  **hybrids** | **Number**  **of Stalks** | **Stalk**  **diameter** | **Plant**  **height** | **Corolla**  **diameter** | **Corolla**  **tube length** | **Corolla**  **tube width** | **Number**  **of flowers** |
| --- | --- | --- | --- | --- | --- | --- | --- |
| **Number of Stalks** | 2,484 | 0,088 | -0,178 | 0,020 | -0,120 | 0,470* | **0,774****** |
| **Stalk diameter** | 0,059 | 0,183 | 0,365 | -0,004 | 0,263 | -0,384 | 0,153 |
| **Plant height** | -1,174 | 0,653 | 17,533 | -0,016 | 0,347 | -0,582** | 0,062 |
| **Corolla diameter** | 0,042 | -0,002 | -0,089 | 1,793 | 0,025 | 0,173 | -0,115 |
| **Corolla tube length** | -0,186 | 0,111 | 1,431 | 0,033 | 0,968 | -0,421 | 0,026 |
| **Corolla tube width** | 0,475 | -0,105 | -1,563 | 0,149 | -0,266 | 0,411 | 0,108 |
| **Number of flowers** | 44,600 | 2,391 | 9,495 | -5,614 | 0,934 | 2,542 | 1337,832 |

| ***E. nevadense***  **parentals** | **Number**  **of Stalks** | **Stalk**  **diameter** | **Plant**  **height** | **Corolla**  **diameter** | **Corolla**  **tube length** | **Corolla**  **tube width** | **Number**  **of flowers** |
| --- | --- | --- | --- | --- | --- | --- | --- |
| **Number of Stalks** | 3,951 | -0,229 | -0,012 | -0,216 | -0,178 | 0,093 | **0,579***** |
| **Stalk diameter** | -0,277 | 0,370 | 0,414* | 0,175 | 0,266 | -0,110 | 0,392* |
| **Plant height** | -0,121 | 1,254 | 24,717 | 0,168 | -0,021 | 0,112 | 0,287 |
| **Corolla diameter** | -0,651 | 0,161 | 1,269 | 2,298 | 0,438** | 0,187 | -0,212 |
| **Corolla tube length** | -0,375 | 0,171 | -0,111 | 0,703 | 1,119 | -0,231 | -0,140 |
| **Corolla tube width** | 0,127 | -0,046 | 0,381 | 0,194 | -0,167 | 0,467 | 0,117 |
| **Number of flowers** | 59,736 | 12,384 | 74,033 | -16,667 | -7,688 | 4,149 | 2692,417 |

**Table S4.** Mean of the Log posterior probabilities (Ln P(K)), their standard deviation (Stdev LnP(K)) (Pritchard et al.2000), and ∆K values (Evanno et al. 2005) for alternative numbers of ancestral genetic clusters (K) as estimated from STRUCTURE.

| K | Reps | Mean LnP(K) | Stdev LnP(K) | ∆K |
| --- | --- | --- | --- | --- |
| 1 | 10 | -5838.21 | 0.5343 | NA |
| 2 | 10 | -5781.12 | 16.3434 | 2.367319 |
| 3 | 10 | -5762.72 | 43.0608 | 0.704121 |
| 4 | 10 | -5714 | 46.3427 | 1.962121 |
| 5 | 10 | -5756.21 | 92.1796 | 0.796054 |
| 6 | 10 | -5725.04 | 85.3463 | NA |
